# Does point of care prothrombin time measurement reduce the transfusion of fresh frozen plasma in patients undergoing major surgery? The POC-OP randomized-controlled trial

**DOI:** 10.1186/1745-6215-10-107

**Published:** 2009-11-23

**Authors:** Natalie Urwyler, Sven Trelle, Lorenz Theiler, Peter Jüni, Lukas P Staub, Cedric Luyet, Lorenzo Alberio, Kay Stricker, Robert Greif

**Affiliations:** 1 University Department of Anesthesiology and Pain Therapy, University Hospital of Bern, Inselspital, 3010 Bern, Switzerland; 2 CTU Bern, Bern University Hospital and Institute of Social and Preventive Medicine, University of Bern, Niesenweg 6, 3012 Bern, Switzerland; 3 NHMRC Clinical Trials Centre, University of Sydney, Locked Bag 77, Camperdown NSW 1450, Australia; 4 University Department of Hematology, University Hospital of Bern, Inselspital, 3010 Bern, Switzerland

## Abstract

**Background:**

Bleeding is a frequent complication during surgery. The intraoperative administration of blood products, including packed red blood cells, platelets and fresh frozen plasma (FFP), is often live saving. Complications of blood transfusions contribute considerably to perioperative costs and blood product resources are limited. Consequently, strategies to optimize the decision to transfuse are needed.

Bleeding during surgery is a dynamic process and may result in major blood loss and coagulopathy due to dilution and consumption. The indication for transfusion should be based on reliable coagulation studies. While hemoglobin levels and platelet counts are available within 15 minutes, standard coagulation studies require one hour. Therefore, the decision to administer FFP has to be made in the absence of any data. Point of care testing of prothrombin time ensures that one major parameter of coagulation is available in the operation theatre within minutes. It is fast, easy to perform, inexpensive and may enable physicians to rationally determine the need for FFP.

**Methods/Design:**

The objective of the POC-OP trial is to determine the effectiveness of point of care prothrombin time testing to reduce the administration of FFP. It is a patient and assessor blind, single center randomized controlled parallel group trial in 220 patients aged between 18 and 90 years undergoing major surgery (any type, except cardiac surgery and liver transplantation) with an estimated blood loss during surgery exceeding 20% of the calculated total blood volume or a requirement of FFP according to the judgment of the physicians in charge. Patients are randomized to usual care plus point of care prothrombin time testing or usual care alone without point of care testing. The primary outcome is the relative risk to receive any FFP perioperatively. The inclusion of 110 patients per group will yield more than 80% power to detect a clinically relevant relative risk of 0.60 to receive FFP of the experimental as compared with the control group.

**Discussion:**

Point of care prothrombin time testing in the operation theatre may reduce the administration of FFP considerably, which in turn may decrease costs and complications usually associated with the administration of blood products.

**Trial registration:**

NCT00656396

## Background

In patients undergoing major non-cardiac surgery there is no established way of monitoring coagulation in real time. In case of intraoperative bleeding, decisions to transfuse fresh frozen plasma (FFP) and procoagulatory drugs have to be taken clinically. Bleeding is a frequent complication during major surgery and can necessitate the transfusion of blood products and/or procoagulatory drugs [1]. Complications of blood transfusions contribute considerably to patient morbidity [2-10] and perioperative costs [11-13]. In patients undergoing major non-cardiac surgery there is no established way of monitoring coagulation during surgery. In case of intraoperative bleeding, decisions to transfuse fresh frozen plasma and procoagulatory drugs are based on clinical judgment only. In view of the risk associated with administration of blood products, the unsatisfactory decision making process and the scarcity of blood products, strategies to optimize intraoperative decision making are much needed.

A patient's bleeding risk may be anticipated based on the patient's diagnosis or on the underlying surgical problem [14-16]. However, the extent of bleeding is not predictable before surgery. Intraoperative bleeding is a dynamic process and major blood loss can occur within minutes with potentially life threatening consequences. Major bleeding can lead to coagulation aberration due to dilution and consumption of coagulation factors [17]. While hemoglobin levels and platelet counts are readily available within 15 minutes, standard coagulation tests have long turn around times of 45 minutes to one hour [18]. Moreover, analyses of specific coagulation factors take up to six hours and results may only be available the next day. These analyses are not performed with emergency priority, some are not even available during night. An intraoperative control of laboratory values is always considered as emergency analysis. Due to the inexistence of intraoperative real time results of standard coagulation studies, the decision to transfuse fresh frozen plasma and procoagulatory drugs during non-cardiac surgery is based on clinical judgment in practice, depending on the experience and/or the subjective decisions of the attending physicians [19].

Point-of-Care (PoC) devices are considerably faster than the standard laboratory methods [20,21]. Several point-of-care tests for standard coagulation parameters and two different systems for thromboelastography are currently marketed. None of the PoC tests, however, is approved for intraoperative real time measurement of coagulation. One potential intra-operative bedside coagulation study system is thromboelastography [22]. It is a viscoelastic test of whole blood clotting, which provides information about the initiation of coagulation, about clot firmness and clot lyses. It is costly and needs trained personnel with a workload of about 15 minutes to get first results. Its use is suggested for cardiac surgery and liver transplantation [23,24]. During major surgery the use of thromboelastography is limited by costs, workload, the need for trained personnel, analysis time, and difficulties in interpreting results.

The use of a coagulation test based transfusion algorithm leads to a reduction in the use of blood products [25,26]. There is no validated, standardized method for measuring intraoperative blood loss. Blood loss during an operation is difficult to quantify [27] and its underestimation might be frequent. Coagulopathic bleeding has to be distinguished from surgical bleeding [28]. Therefore coagulation parameters must be measured intraoperatively when major bleeding occurs and decisions for or against the transfusion of blood products and procoagulatory drugs have to be made [1]. The availability of coagulation tests is crucial for any algorithm used for decision making on intra-operative substitution of blood products.

PoC devices could be used in the intraoperative setting to guide the use of the costly blood bank resources and/or procoagulatory drugs. In this study we will use a transfusion algorithm based on the ASA Guidelines [1,12] and point of care measurement of prothrombin time.

## Methods/Design

### Objective

To determine the effectiveness of an intraoperative decision algorithm based on PoC testing of prothrombin time in reducing the administration of fresh frozen plasma.

### Study design

**Figure 1:**
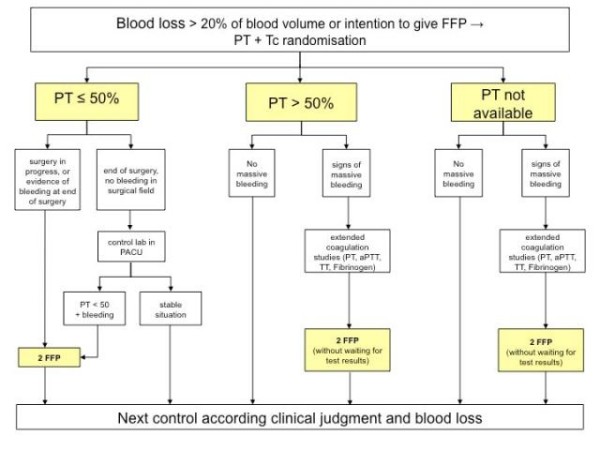
**Algorithm for the transfusion of fresh frozen plasma**. FFP = Fresh Frozen Plasma, PT = Prothrombin Time, Tc = Thrombocyts, PACU = Post Anesthesia Care Unit, aPTT = activated Partial Tromboplstine Time, TT = Thrombin Time.

**Figure 2:**
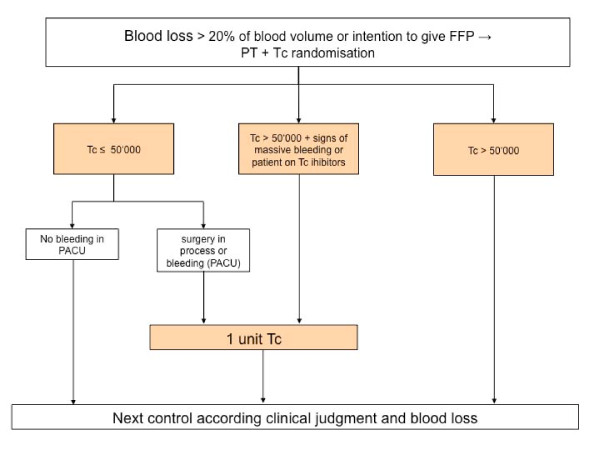
**Algorithm for the transfusion of platelets**. FFP = Fresh Frozen Plasma, PT = Prothrombin Time, Tc = Thrombocyts, PACU = Post Anesthesia Care Unit.

The POC-OP trial is an ongoing, patient and assessor blind, parallel-group, single-center randomized controlled trial. Patients are randomized to (1) current clinical treatment and perioperative care plus point of care prothrombin time testing or (2) current clinical treatment and perioperative care without point of care testing. Trials evaluating a combination of test strategies and subsequent treatments require a pre-specified link between test results and management decisions [29]. In the POC-OP trial, the treatment options and their relation with the different PoC test results are specified in the algorithm (figure 1 and figure 2).

### Patient population

Men and women of at least 18 years of age undergoing major surgery (orthopedic surgery, trauma surgery, neuro-surgery, plastic and reconstructive surgery, ear-nose-throat surgery, urologic surgery, gynecologic surgery, thoracic surgery, visceral surgery) are eligible if their preoperative estimated blood loss during surgery exceeds 20% of the individually calculated normal total blood volume or if the treating anesthesiologist or surgeon intends to give fresh frozen plasma. Surgical management of platelet aggregation inhibitors changed in 2007 for patients with cardiac diseases and/or coronary artery stents [30]. Therefore, we will consider all patients scheduled for operation, even if anticoagulants or anti-platelet drugs were administered.

Patients are not eligible if they have a history of hereditary coagulopathies; are pregnant; have abnormal coagulation studies before surgery (based on normal values for prothrombin time: 70 - 130% and platelets: 140 - 380 G/l); undergo liver transplantation; or patients receiving a bolus of heparin intraoperatively (extracorporal circulation 400 - 500 IU heparin per kg bodyweight, vascular surgery 100 IU before clamping the artery). Prothrombin time in patients under unfractionated heparin > 1 IU per ml blood can impair the accuracy of the result of our PoC device [31]. Liver transplantation might pose a more complex problem for coagulation than dilution or consumption coagulopathy only [32]. During liver transplantation, coagulation should be monitored by rotation thrombelastometry [23]. PoC prothrombin time monitoring might be helpful but not sufficient for complex coagulation aberrations. Anesthetic management (general-, regional or a combined anesthesia) is defined by the attending anesthesiologist. Prothrombin time is not influenced by anesthetic gases, propofol, opiates, muscle relaxants and local anesthetics [33,34]. Propofol, desflurane, sevoflurane or isoflurane are used for general anesthesia in our Department. Regional Anesthesia is only performed in patients with normal preoperative coagulation studies.

### Allocation of patients

**Figure 3:**
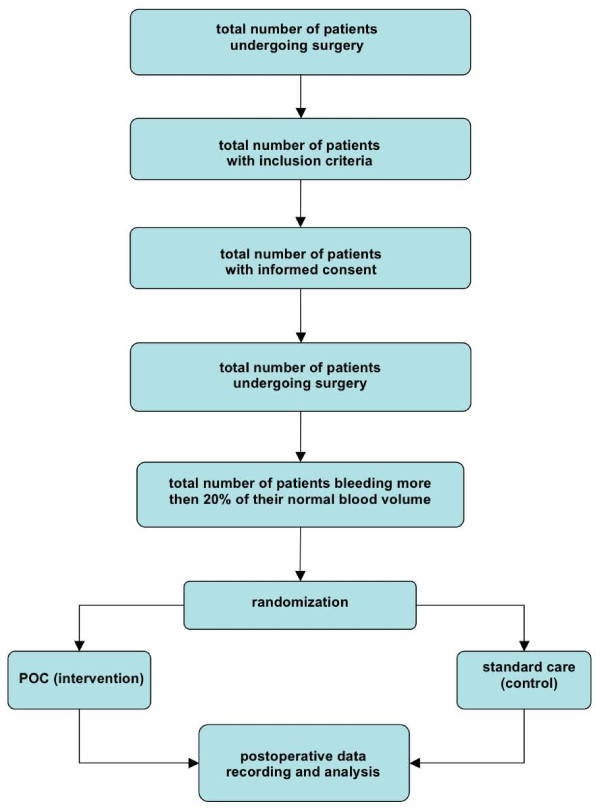
**Study flow for individual patients**.

Informed consent of eligible patients is obtained on the day before surgery. Patients with written informed consent are randomly allocated to the experimental and control intervention only if their intra-operatively estimated blood loss exceeds 20% of the patients calculated normal total blood volume (70 ml per kg body weight) or if their treating anesthesiologist or surgeon intends to give fresh frozen plasma (Figure 3).

Allocation is done in a 1:1 fashion based on a computer-generated random list, with randomly varying blocks of four and six. Sealed, opaque, sequentially numbered, tamper-proof security envelopes are used to conceal allocation. Preparation of envelopes was done by personnel independent of patient recruitment.

### Perioperative care

An attending anesthesiologist is in charge of all decisions regarding anesthesia and transfusion of blood products. Residents in anesthesiology work continuously under supervision of the attending physician. During the trial, the attending anesthesiologist ensures that the trial protocol is followed properly.

When the intraoperative estimated blood loss exceeds 20% of the individuals total blood volume, or if their treating anesthesiologist or surgeon intends to give fresh frozen plasma, patients are randomized as described above and patient management determined according to the group assignment.

All patients receive allogenic blood products according to pre-defined transfusion algorithms (Figure 1 and Figure 2). Both algorithms were developed based on a literature review [1,12,13] and recommendations by in-house hematologists.

The landmarks of the algorithms are:

- Blood loss of more than 20%

- Clinical assessment of bleeding type, i.e. massive or not by the attending physicians

- Prothrombin time of less than 50%

- Platelet count of less than 50.000 per liter

Blood samples for all coagulation studies are drawn from arterial and central venous lines, peripheral venous lines, by direct venipuncture or capillary, according to the standard operating procedure of the University Department of Anesthesiology and Pain Therapy of the University Hospital Bern. Prothrombin time is measured according to the randomization and transfusion of fresh frozen plasma based on available results of the prothrombin time test and clinical judgment (Figure 1). For both groups, platelet counts are done in the central hematology laboratory of the University Hospital Bern and thrombocytopenia is treated according to the algorithm (Figure 2). Hemoglobin is measured PoC in the operating room by Hemocue (B-Hemoglobinphotometer Hemocue^® ^AB, Ängelholm, Sweden) and/or in the central hematology laboratory. In case of anemia, erythrocytes are transfused according to the standard operating procedure of the Department of Anesthesiology based on ASA guidelines (1). Platelet counts and hemoglobin levels as measured by the central hematology laboratory are usually available within 15 minutes. The central hematology laboratory has an ISO/IEC 17025 accreditation [35].

Postoperative care is the same for both groups. Pain treatment consists of continuous regional anesthesia, opioids either continuous or per bolus, as well as paracetamol, metamizol and NSAIDs.

### Experimental intervention

According to the transfusion algorithm (Figure 1) bedside prothrombin time (PT) is measured by a PoC device (CoaguChek XS Plus^® ^Roche Diagnostics, Basel, Switzerland) and immediately available for decision-making. We have demonstrated the high diagnostic accuracy of CoaguChek in a previous study. [36] Of note, agreement for prothrombin time values between capillary and venous or arterial blood was evaluated and showed good agreement [37].

### Control intervention (standard care)

Patients in the control group are transfused according to the same algorithms and triggers as in the experimental group. However, in the absence of the PoC device, prothrombin time is measured in the central hematology laboratory by BCS coagulation systems (BCS, Siemens Healthcare Diagnostics, USA). Considering a turn around time which usually exceeds 40 minutes [18], the results of intra-operative prothrombin time measurements in the control group are usually not available for decision-making. Decisions for a transfusion of fresh frozen plasma in the standard care group are therefore based on clinical judgment only.

### Emergencies

In case of life threatening rapid massive blood loss with no time to take blood samples for coagulation studies, the anesthesiologist is allowed to deviate from the protocol at his or her discretion.

### Assessments

The following assessments are performed for all randomized patients regardless of any protocol deviations:

#### Preoperative

Patient's demographics, American Society of Anesthesiology physical health status classification, coagulation history, surgical diagnosis, other secondary diagnosis, drugs with influence on coagulation or platelet function (coumarine-derivatives, platelet aggregation inhibitors, unfractionated heparin, low molecular weight heparins, non-steroidal anti-inflammatory drugs), time when these drugs were stopped, other long-term medication, surgical discipline, planed operation, planed anesthesia, preoperative hemoglobin level, prothrombin time and platelet count.

#### Post-operative

Type and duration of operation; type and duration of anesthesia; intraoperative blood loss, transfused blood products, amount and type of i.v. fluids, procoagulatory drugs and amount, laboratory studies, serious intra-operative adverse events, life threatening bleeding resulting in life-saving measures and consecutive protocol violations.

#### First post-operative day

Transfused blood products since end of surgery, amount and type of i.v. fluids, procoagulatory drugs, laboratory values at the end of surgery and 24-h later: laboratory studies. Duration of stay in post-anesthesia care unit in hours, duration of stay in ICU in hours, reoperation due to bleeding, serious adverse events, survival.

#### Hospital discharge or 7st day post operation

Transfused blood products, procoagulatory drugs, duration of stay in post anesthesia care unit in hours, duration of stay in intensive care unit in hours, duration of hospitalization since randomization, reoperation due to bleeding, serious adverse events, vital status.

#### Follow-up at 6 months

Re-hospitalization and days of hospitalization, reoperation due to bleeding, serious adverse events, vital status.

### Outcomes

The primary outcome of the trial is:

1) the relative risk of receiving any fresh frozen plasma in-hospital, defined as the administration of at least 0.5 units of fresh frozen plasma received after randomization until day 7 after surgery or post-operative discharge from hospital, whatever comes first.

Secondary outcomes are:

2) the number of units of fresh frozen plasma received after randomization until day 7 or post-operative discharge from hospital, whatever comes first;

3) the number of units of allogenic blood products transfused

4) the relative risk of major in-hospital bleeding defined as bleeding event requiring an extension of hospitalization, reoperation due to bleeding, bleeding resulting in hemorrhagic shock or death after randomization until day 7 or post-operative discharge from hospital, whatever comes first;

5) the relative risk of a serious adverse event (non-fatal myocardial infarction, non-fatal stroke, cardiovascular death, or death of unknown cause) [38] received after randomization until day 7 or post-operative discharge from hospital, whatever comes first;

6) the relative risk of overall mortality received after randomization until day 7 or post-operative discharge from hospital, whatever comes first.

Other exploratory outcomes of interest are: the estimated extent of blood loss, the relative risk of a serious adverse events; the relative risk of a reoperation because of a bleeding complication; the relative risk of receiving any allogenic blood product; the relative risk of a systemic inflammatory response syndrome (SIRS) or sepsis; the relative risk of an infection of the surgical wound; the compliance with the allocated intervention.

In-hospital outcomes are ascertained by study personnel unrelated to the care of randomized patients and 6-month data is recorded by telephone interviewers. Patients, study personnel recording outcome data in-hospital and telephone interviewers ascertaining 6-month data are blind as to the allocated intervention.

### Sample size

The POC-OP trial is a superiority trial. A review of 40 randomly selected patients in our institution with major orthopedic surgery and an intraoperative blood loss of at least 20% revealed that 50% of these patents received fresh frozen plasma. Consequently, a baseline risk of 50% of receiving fresh frozen plasma was assumed for the sample size calculation. We defined a relative risk of 0.60 of receiving fresh frozen plasma as minimally clinically relevant relative risk. A sample size of 110 patients per study arm will provide more than 80% power to detect a relative risk of 0.60 at a two-sided alpha level of 5%.

### Statistical analysis

The trial statistician will be blinded to the allocated intervention for all analyses.

All analyses will be based on the intention-to-treat principle, whereby all randomized patients will be included in the analysis in the group they were allocated regardless of the intervention received.

Binary data will be analyzed using the chi-squared test. Continuous data will be analyzed using Student's t-test. Missing values will be imputed using multiple imputation [39]. For stratified analyses of the primary outcome we will use formal tests of interaction between allocated interventions and subgroup in multivariable logistic regression models [40]. The following stratification factors will be analyzed: PT ≥ 90% versus PT < 90% at baseline; trauma/emergency versus elective surgery; orthopedic versus other surgeries; American Society of Anesthesiology physical health status classification ≥ 4 versus American Society of Anesthesiology physical health status classification < 4.

### Ethical approval

The POC-OP randomized-controlled trial has been approved by the ethic committee of the canton of Bern (41), ref. nr.: 232/06

### Quality assurance

Source data verification of selected data is performed by an independent monitor of CTU Bern in a random sample of 40 patients. The following data is monitored: eligibility criteria, hemoglobin at baseline, American Society of Anesthesiology physical health status classification status, compliance with allocated intervention. In case of discrepancies in more than 14 per 1000 data points, we will perform a source-data verification in all patients. Written informed consent is verified by the monitor in all patients.

## Discussion

The transfusion of blood products can be live saving, but carries substantial side effects for the patient [2-10]. Therefore, the indication for the administration of allogenic blood products has to be weighed up against the risks for the patient. Also, blood products are a scarce resource necessitating their efficient use. Until today there is no satisfying option to monitor the dynamic process of intraoperative bleeding without considerable delay and high costs.

The study project is designed to evaluate a simple method, which ensures that results of coagulation studies become available to the anesthesiologist in charge without delay. To reflect modern concepts of decision making, we created a transfusion algorithm, which follows ASA guidelines [1,12]. PoC testing in the operation theatre in combination with this algorithm may reduce the administration of fresh frozen plasma considerably, which in turn may decrease costs and complications usually associated with the administration of allogenic blood products.

## List of Abbreviations

POC-OP: Point Of Care in the Operating Room; FFP: Fresh Frozen Plasma; PoC: Point-of-Care; ASA: American Society of Anesthesiologists; Tc: Thrombocytes; PT: Prothrombin Time; PACU: Post Anesthesia Care Unit; ICU: Intensive Care Unit; aPTT: activated Partial Thromboplastine Time; TT: Thrombin Time; NSAID: Non Steroidal Anti Inflammatory Drug.

## Competing interests

We receive the PoC devices, test stripes, quality controls and the training for the study personnel from Roche Diagnostics without costs. Roche will be in charge for serial quality controls of the PoC devices.

## Authors' contributions

NU is the Principle Investigator for the study, contributed to the study design and to drafting and revising the manuscript. ST made significant contributions to concept of the study, drafting and reviewing manuscript. LT was responsible for the organization of data acquisition and staff training in the operational theatre. He assisted in revising and proof reading the manuscript. PJ made significant contributions to the concept of the study and reviewing the manuscript. LS participated in developing the concept of the study, sample size estimation, drafting, reviewing and proof reading the manuscript. CL was involved in the organization of data acquisition and staff training in the operational theatre. LA participated in the design of the algorithm as a hematological consultant. KS is responsible for material, machines and drugs in our institution. He assisted in choosing and evaluating the point of care tool for our study in cooperation with the department of hematology (LA). RG participated in developing the concept of the study, drafting reviewing the manuscript. All authors read and approved the final manuscript.
